# Exercise training results in depot-specific adaptations to adipose tissue mitochondrial function

**DOI:** 10.1038/s41598-020-60286-x

**Published:** 2020-03-02

**Authors:** Amy E. Mendham, Steen Larsen, Cindy George, Kevin Adams, Jon Hauksson, Tommy Olsson, Melony C. Fortuin-de Smidt, Pamela A. Nono Nankam, Olah Hakim, Louise M. Goff, Carmen Pheiffer, Julia H. Goedecke

**Affiliations:** 1 Non-communicable Diseases Research Unit, South African Medical Research Council, Cape Town, South Africa; 2 Division of Exercise Science and Sports Medicine, Department of Human Biology, University of Cape Town, Cape Town, South Africa; 3 Center for Healthy Aging, Department of Biomedical Sciences, Copenhagen University, Copenhagen, Denmark; 4 Clinical Research Centre, Medical University of Bialystok, Bialystok, Poland; 5 Department of Radiation Sciences, Radiation Physics and Biomedical Engineering, Umeå University, Umeå, Sweden; 6 Department of Public Health and Clinical Medicine, Umeå University, Umeå, Sweden; 7 Department of Diabetes, School of Life Course Sciences, Faculty of Life Sciences and Medicine, King’s College London, London, UK; 8 Biomedical Research and Innovation Platform, South African Medical Research Council, Cape Town, South Africa

**Keywords:** Fat metabolism, Obesity, Pre-diabetes, Mitochondria

## Abstract

We assessed differences in mitochondrial function in gluteal (gSAT) and abdominal subcutaneous adipose tissue (aSAT) at baseline and in response to 12-weeks of exercise training; and examined depot-specific associations with body fat distribution and insulin sensitivity (S_I_). Obese, black South African women (n = 45) were randomized into exercise (n = 23) or control (n = 22) groups. Exercise group completed 12-weeks of aerobic and resistance training (n = 20), while the control group (n = 15) continued usual behaviours. Mitochondrial function (high-resolution respirometry and fluorometry) in gSAT and aSAT, S_I_ (frequently sampled intravenous glucose tolerance test), body composition (dual-energy X-ray absorptiometry), and ectopic fat (MRI) were assessed pre- and post-intervention. At baseline, gSAT had higher mitochondrial respiratory capacity and hydrogen peroxide (H_2_O_2_) production than aSAT (p < 0.05). Higher gSAT respiration was associated with higher gynoid fat (p < 0.05). Higher gSAT H_2_O_2_ production and lower aSAT mitochondrial respiration were independently associated with lower S_I_ (p < 0.05). In response to training, S_I_ improved and gynoid fat decreased (p < 0.05), while H_2_O_2_ production reduced in both depots, and mtDNA decreased in gSAT (p < 0.05). Mitochondrial respiration increased in aSAT and correlated with a decrease in body fat and an increase in soleus and hepatic fat content (p < 0.05). This study highlights the importance of understanding the differences in mitochondrial function in multiple SAT depots when investigating the pathophysiology of insulin resistance and associated risk factors such as body fat distribution and ectopic lipid deposition. Furthermore, we highlight the benefits of exercise training in stimulating positive adaptations in mitochondrial function in gluteal and abdominal SAT depots.

## Introduction

Adipose tissue is recognised as an endocrine organ with different depots showing differences in cell morphology, gene expression, function and overall disease risk^1–5^. The accumulation of abdominal adipose tissue is closely linked to insulin resistance^6–8^. Specifically, the excess accumulation of visceral adipose tissue (VAT) and ectopic lipids are associated with reduced insulin sensitivity (S_I_)^6,7,9^, while the storage of fat in peripheral subcutaneous adipose tissue (SAT) depots is associated with an insulin sensitive phenotype^6^. Notably, ethnic differences in body fat distribution have been reported, with black African women presenting with a phenotype of relatively lower VAT and high peripheral SAT that is associated with reduced S_I,_ when compared to their white counterparts^1,10^. The reason for this paradox is not clear. We suggest that depot-specific mitochondrial functions may be a key mediator.

The plasticity of the mitochondria dictates the adipocytes’ ability to handle excess lipids and changes in energy demand. In particular, obesity and the development of type 2 diabetes is associated with impaired mitochondrial function in adipocytes, which can be defined by a reduction in β-oxidation that leads to increased cytosolic free fatty acids, altered glucose uptake and increased triglyceride synthesis^11,12^. Importantly, hyperglycaemia and the excess supply of fatty acids and lipid accumulation in the cell increases the inflammatory profile and ROS production, which further impairs mitochondrial function^13^. Mitochondrial function encompasses cellular oxygen consumption (respiration) and hydrogen peroxide (H_2_O_2_) emission. In adipocytes, mitochondrial respiration reflects the cells’ oxidative capacity and substrate metabolism^2^. Moreover, the emission rate of H_2_O_2_ incorporates the production and clearance by the antioxidant system and represents the balance between electron leak and superoxide formation from cellular respiration and modulates the overall cellular redox environment^14^. Previously, the gluteal SAT (gSAT) depot in obese African women was shown to have a higher inflammatory profile when compared to abdominal SAT (aSAT)^1^; however, differences in mitochondrial function between aSAT and gSAT depots, and their relationship with whole-body S_I_ and body fat distribution have not been previously explored in humans. Exercise training has been shown to reduce abdominal fat (VAT and SAT), and ectopic lipid deposition (i.e. liver, skeletal muscle and pancreas), and improve S_I_^15–19^. These adaptations in fat deposition may infer differences between depots in mitochondrial function when responding to changes in energy demand. However, no study to our knowledge has investigated the role of mitochondrial function within adipose tissue in mediating these exercise-induced changes in humans. Our hypothesis was that a difference in mitochondrial function between aSAT and gSAT depots at baseline will result in depot-specific adaptations to exercise training and associate with changes in body fat distribution and S_I_. Accordingly, this study aimed to: i) compare mitochondrial function (respiration and H_2_O_2_ emissions) and gene expression in aSAT and gSAT at baseline; ii) investigate depot-specific adaptions in mitochondrial function and gene expression in response to 12-weeks of exercise training; and iii) assess depot-specific associations between mitochondrial function and body fat distribution and whole-body S_I_ at baseline and in response to the exercise intervention.

## Results

### Compliance, physical activity and dietary behaviors

Details on recruitment and the sociodemographic characteristics of participants are reported in *Goedecke et al*.^20^. Forty-five participants completed baseline testing, and participants that completed the 12-week intervention included n = 20 in the exercise group and n = 15 in the control group. Of the 48 exercise sessions conducted, participants attended 79 ± 13 (range: 52–100)% at a mean intensity of 79.7 ± 4.0 (range: 71–85) % peak heart rate (HR_peak_). Daily energy expenditure (reported as kJ/day and METs h/day) and energy consumption (reported as kJ/day and relative (%) macronutrient consumption) did not differ within or between groups at baseline and at 4, 8 and 12 weeks (All p > 0.05; Supplementary Table 1).

### Cardiorespiratory fitness, body composition, S_I_ and ectopic lipids in response to the intervention

Differences in cardiorespiratory fitness, body composition, S_I_ and ectopic lipids at baseline and in response to the 12-week intervention are presented in brief in Table 1. There was a small but significant decrease in body mass index (BMI) in the exercise group and increase in the control group (P = 0.003 for interaction). Exercise training also resulted in increased peak oxygen consumption (VO_2peak_; p < 0.001 for interaction) and S_I_ (p = 0.037 for interaction), and although not clinically significant, there was a small reduction in gynoid fat mass (%FM) (p = 0.002 for interaction), that were not observed in the control group. In contrast, abdominal SAT volume increased in the control group, with no change in the exercise group (p = 0.018 for interaction).

**Table 1:** Cardiorespiratory fitness, S_I,_ body composition and ectopic lipids at baseline and in response to the 12-week intervention.

Variable	EXERCISE (n = 20)		CONTROL (n = 15)		Group	Time	Interaction
	Pre	Post	Pre	Post	P Value	P Value	P Value
Age (y)	22 (21, 24)	—	23 (21, 27)	—			
VO_2peak_ (mL/kg/min)	24.9 ± 2.4	27.6 ± 3.4	23.9 ± 2.8	22.9 ± 2.6	0.291	0.195	**<0.001**
VO_2peak_ (mL/min)	2078 ± 211	2278 ± 231	2099 ± 282	2032 ± 196	0.447	0.144	**<0.001**
S_I_ (mU/L)/min	2.0 (1.2, 2.8)	2.2 (1.1, 3.3)*	2.0 (0.8, 3.2)	1.8 (1.2, 2.4)	0.094	0.711	**0.037**
***Anthropometry***							
Weight (kg)	84.1 ± 8.7	83.3 ± 9.7*	87.8 ± 10.9	88.8 ± 11.0*	0.267	**0.030**	**0.003**
BMI (kg/m^2^)	34.1 ± 2.8	33.8 ± 3.1*	33.4 ± 2.7	33.8 ± 2.8*	0.430	**0.038**	**0.003**
Waist-to-hip ratio (AU)	0.89 (0.87, 0.94)	0.87 (0.86, 0.91)	0.88 (0.83, 0.93)	0.90 (0.84, 0.96)	0.629	0.804	**0.022**
***Dual-Energy X-ray Absorptiometry***							
FFSTM (kg)	37.1 (33.5, 39.5)	37.1 (33.7, 39.9)	37.7 (34.6, 40.8)	38.2 (35.2, 40.9)	0.293	0.223	0.324
Fat Mass (kg)	38.6 ± 5.5	38.6 ± 6.7	40.3 ± 7.0	41.2 ± 6.2	0.302	0.076	0.189
Fat Mass (%)	49.9 (48.5, 51.6)	49.9 (48.3, 51.0)	49.8 (46.7, 52.7)	50.9 (47.7, 52.9)	0.981	0.480	0.471
Android FM (%FM)	8.3 ± 1.0	8.1 ± 1.1	8.0 ± 1.4	7.9 ± 1.5	0.572	0.163	0.860
Gynoid FM (%FM)	18.5 ± 1.7	18.2 ± 1.6*	19.5 ± 2.3	19.6 ± 2.3	0.129	0.323	**0.002**
***MRI***							
VAT (cm^3^)	920.0 ± 322.1	906.2 ± 346.9	884.5 ± 444.7	925.8 ± 409.2	0.850	0.177	0.178
Abdominal SAT (cm^3^)	5489.3 ± 1053.7	5447.7 ± 1260.7	5277.1 ± 1934.5	5580.7 ± 2043.9*	0.850	**0.008**	**0.018**
Pancreatic fat (%)	7.8 (6.4, 10.4)	6.9 (5.5, 8.3)	7.0 (5.2, 8.0)	6.5 (5.6, 7.0)	0.476	0.209	0.455
Hepatic fat (%)	4.9 (4.5, 6.4)	4.8 (3.9, 5.6)	4.7 (4.3, 5.3)	4.7 (4.2, 6.2)	0.211	0.487	0.103
Tibialis Anterior fat (%)	5.0 (2.9, 6.3)	4.2 (3.3, 5.4)	3.4 (2.7, 3.9)	4.0 (32.7, 4.7)	0.674	0.979	0.554
Soleus fat (%)	10.4 (7.4, 12.7)	9.9 (8.4, 11.2)	9.7 (8.5, 10.8)	9.5 (8.6, 11.3)	0.478	0.682	0.522

Data reported as mean ± Standard Deviation for normally distributed variables and as median (25–75%, Interquartile Range) for skewed variables. Mixed-model analyses identified main effects of time (pre and post) and group (exercise and control), and interaction (group x time) effects reported for exercise (n = 20) and control (n = 15) groups. Significant change within the group, p < 0.05*. BMI, Body Mass Index; VO_2peak_, Peak Oxygen Consumption; S_I,_ Insulin Sensitivity; FFSTM, Fat-Free Soft Tissue Mass; %FM, percent of total fat-mass; VAT, Visceral Adipose Tissue; SAT, Subcutaneous Adipose Tissue.

### Baseline comparisons in mitochondrial function, gene and protein expression between abdominal and gluteal SAT depots

**Figure 1:**
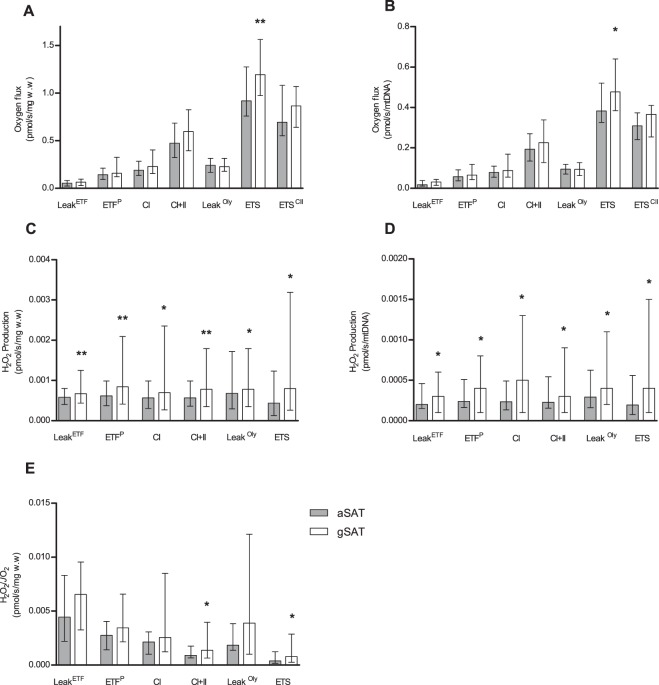
Baseline comparisons of mitochondrial respiration (**A,B**) and H_2_O_2_ production (**C**–**E**) between abdominal and gluteal subcutaneous adipose tissue. All data is reported as median (25–75% Interquartile Range). Paired t-tests identified differences at baseline between depots (n = 37). Significant difference between abdominal and gluteal depots *p < 0.05; **p < 0.001. Leak^ETF^, leak respiration through electron-transferring flavoprotein; ETF^P^, Lipid oxidative phosphorylation capacity; CI, Complex 1 linked respiration; CI + CII, Complex 1 and 2 linked respiration (oxidative phosphorylation capacity); Leak^Oly^, Oligomycin (ATP synthase inhibitor) linked leak respiration; ETS, Electron transfer system capacity; ETS^II^, Complex 2 linked electron transfer system capacity.

At baseline, mtDNA did not differ between depots (p = 0.695; Supplementary Table 2). Figure 1 shows baseline comparisons of mitochondrial respiration and H_2_O_2_ production between aSAT and gSAT, expressed relative to wet weight (w.w) and mitochondrial DNA (mtDNA). The adjustment in w.w and mitochondrial content (mtDNA) represents total tissue function and intrinsic mitochondrial function, respectively. Furthermore, H_2_O_2_ production is dependent on the respiratory capacity of the mitochondria and is therefore also reported relative to oxygen flux. Mitochondrial respiration (w.w and mtDNA-adjusted) had a higher electron transport system (ETS) capacity in gSAT compared to aSAT (p = 0.043, Fig. 1A,B). Further, H_2_O_2_ production (w.w and mtDNA-adjusted) during all states of respiration were significantly higher in the gSAT compared to aSAT (p < 0.05, Fig. 1C, D); however, when adjusted for oxygen flux, H_2_O_2_ production was significantly higher in gSAT during only CI + II and ETS respiratory states (p < 0.05; Fig. 1E). The respiratory control ratios, which reflect differences in mitochondrial coupling, did not differ between depots at baseline (p > 0.05, Table 2). Adipose triglyceride lipase (ATGL) was the only gene to differ between depots at baseline (Supplementary Table 2), with higher expression in gSAT compared to aSAT (p = 0.016).

**Table 2:** Adipose tissue mitochondrial respiration ratios pre and post 12-week intervention.

Variable	BASELINE	EXERCISE		CONTROL		Time	Group	Interaction
		Pre	Post	Pre	Post	P Value	P Value	P Value
***Abdominal Subcutaneous Adipose Tissue***								
CI/CI + CII	0.43 ± 0.12	0.40 ± 0.14	0.41 ± 0.15	0.45 ± 0.12	0.39 ± 0.13	0.475	0.539	0.265
ETF^P^/CI	0.77 (0.65, 0.84)	0.76 (0.66, 0.86)	0.68 (0.56, 0.81)	0.79 (0.74, 0.84)	0.75 (0.68, 0.83)	0.285	0.086	0.627
ETF^P^/CI + CII	0.32 ± 0.11	0.28 ± 0.10	0.28 ± 0.13	0.35 ± 0.11	0.29 ± 0.13	0.265	0.147	0.670
Leak^ETF^/CI	0.24 (0.14, 0.43)	0.26 (0.11, 0.42)	0.11 (0.07, 0.15)	0.24 (0.09, 0.40)	0.19 (0.09, 0.29)	**<0.001**	0.225	0.108
Leak^ETF^/CI + CII	0.08 (0.06, 0.18)	0.08 (0.03, 0.14)	0.04 (0.02, 0.06)	0.13 (0.04, 0.22)	0.09 (0.06, 0.12)	**0.001**	0.196	0.367
***Gluteal Subcutaneous Adipose Tissue***								
CI/CI + CII	0.45 ± 0.10	0.45 ± 0.10	0.49 ± 0.14	0.44 ± 0.12	0.50 ± 0.08	0.124	0.776	0.556
ETF^P^/CI	0.76 ± 0.19	0.78 ± 0.21	0.73 ± 0.12	0.73 ± 0.20	0.76 ± 0.09	0.835	0.741	0.305
ETF^P^/CI + CII	0.34 ± 0.12	0.35 ± 0.11	0.36 ± 0.13	0.33 ± 0.14	0.38 ± 0.10	0.224	0.987	0.280
Leak^ETF^/CI	0.25 (0.14, 0.44)	0.35 ± 0.19	0.25 ± 0.19	0.27 ± 0.20	0.21 ± 0.15	0.056	0.287	0.791
Leak^ETF^/CI + CII	0.11 (0.06, 0.20)	0.18 (0.12, 0.26)	0.09 (0.01, 0.17)	0.08 (0.05, 0.12)	0.10 (0.03, 0.17)	0.112	0.364	0.670

Normally distributed data reported as Mean ± Standard Deviation and data not normally distributed reported as Median (25–75% Interquartile Range). Paired t-tests identified differences at baseline between depots (n = 37). Mixed-model analyses identified main effects of time (pre and post) and group (exercise and control), and interaction (group x time) effects in exercise (n = 19, both depots) and control (n = 14 in abdominal and n = 13 in gluteal depots) groups. Leak^ETF^, leak respiration through electron-transferring flavoprotein; ETF^P^, Lipid oxidative phosphorylation capacity; CI, Complex 1 linked respiration; CI + CII, Complex 1 and 2 linked respiration (oxidative phosphorylation capacity).

### Depot-specific adaptations in mitochondrial function, protein and gene expression in response to the exercise intervention

**Figure 2:**
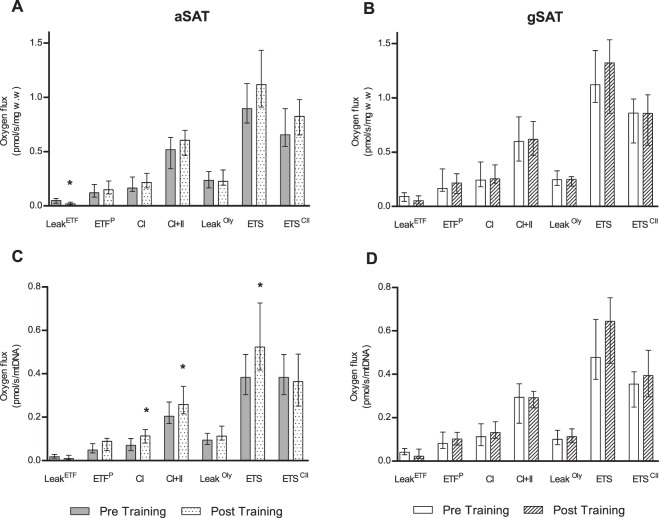
Change in mitochondrial respiration in response to a 12-week exercise training intervention. (**A,C**) represent change in abdominal subcutaneous adipose tissue (aSAT). (**B,D**) represent change in gluteal SAT (gSAT). All data reported as median (25–75% Interquartile Range). Mixed-model analyses identified main time (pre and post), group (exercise and control), and interaction (group x time) effects in exercise (n = 19, both depots) and control (n = 14 in abdominal and n = 13 in gluteal depots) groups. Significant difference between pre and post exercise training *p < 0.05. Leak^ETF^, leak respiration through electron-transferring flavoprotein; ETF^P^, Lipid oxidative phosphorylation capacity; CI, Complex 1 linked respiration; CI + CII, Complex 1 and 2 linked respiration (oxidative phosphorylation capacity); Leak^Oly^, Oligomycin (ATP synthase inhibitor) linked leak respiration; ETS, Electron transfer system capacity; ETS^II^, Complex 2 linked electron transfer system capacity.

Changes in mitochondrial respiration (expressed relative to w.w and mtDNA) in response to exercise training are shown in Fig. 2, with control data reported in Supplementary Fig. 1. A decrease in gSAT mtDNA (Supplementary Table 1; p = 0.044 for post-hoc) occurred in response to exercise training, with no changes in aSAT (P > 0.05). In aSAT, there was a significant reduction in w.w adjusted Leak^ETF^, however when adjusted for mtDNA there was no change in Leak^ETF^, but rather a significant increase in CI (p = 0.009), CI + II (p = 0.013) and ETS capacity (p = 0.008) respiratory states in response to the exercise training (Fig. 2C). Further, improved mitochondrial coupling in response to the exercise training was observed in aSAT only, and was reflected by a decrease in aSAT leak^ETF^/CI (p < 0.001) and Leak^ETF^/CI + II (p = 0.002) respiratory control ratios (Table 2). In contrast, no changes in mitochondrial respiration (Fig. 2B,D) or respiratory control ratios (Table 2) were reported for gSAT. In the control group (Supplementary Fig. 1), ETF^P^ (p = 0.027) and CI (p = 0.048) respiratory states (mtDNA-adjusted) increased in only the gSAT and CI + II increased in the aSAT depot (p = 0.050).

**Figure 3:**
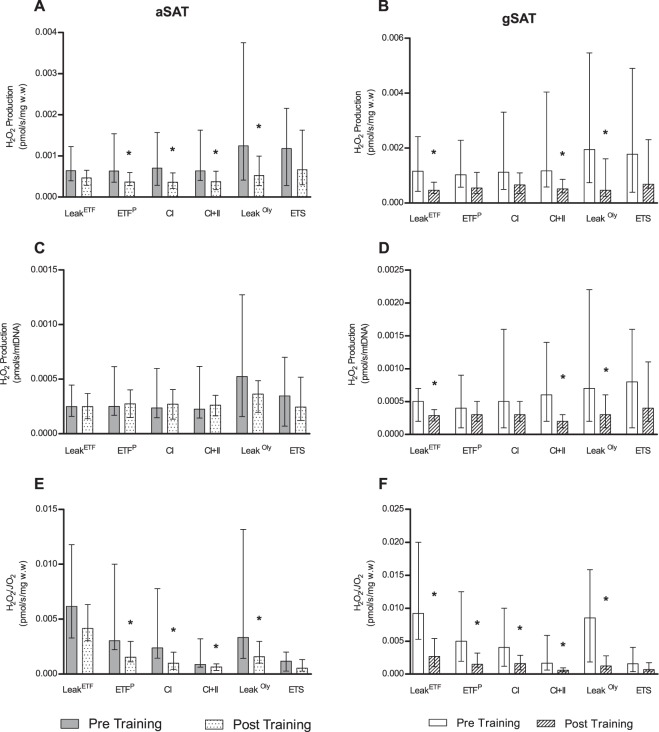
Change in mitochondrial H_2_O_2_ production in response to a 12-week exercise training intervention. (**A,C,E**) represent change in abdominal subcutaneous adipose tissue (aSAT). (**B,D,F**) represent change in gluteal SAT (gSAT). Mixed-model analyses identified main time (pre and post), group (exercise and control), and interaction (group x time) effects in exercise (n = 19, both depots) and control (n = 14 in abdominal and n = 13 in gluteal depots) groups. All data reported as median (25–75% Interquartile Range). Significant difference between pre and post exercise training *p < 0.05. Leak^ETF^, leak respiration through electron-transferring flavoprotein; ETF^P^, Lipid oxidative phosphorylation capacity; CI, Complex 1 linked respiration; CI + CII, Complex 1 and 2 linked respiration (oxidative phosphorylation capacity); Leak^Oly^, Oligomycin (ATP synthase inhibitor) linked leak respiration; ETS, Electron transfer system capacity.

Depot-specific changes in H_2_O_2_ production (expressed relative to w.w, mtDNA and oxygen flux) for the exercise group are shown in Fig. 3, with control data presented in Supplementary Fig. 2. In aSAT, H_2_O_2_ production, adjusted for w.w and oxygen flux, was reduced in ETF^P^, CI, CI + II, leak^oly^ respiratory states in response to exercise training (p < 0.05; Fig. 3A,E). However, when adjusting for mtDNA, these differences were no longer significant (p > 0.05; Fig. 3C). In gSAT, H_2_O_2_ production (using all adjustments) was reduced in leak^ETF^, CI + II and leak^oly^ respiratory states (p < 0.05; Fig. 3B,D,F), with ETF^p^ and CI, also being reduced in response to the exercise training when adjusting for only oxygen flux (p < 0.05; Fig. 3F). No changes were observed in H_2_O_2_ production during all states of respiration in the control group (Supplementary Fig. 2; p > 0.05).

Changes in gene expression in response to the intervention are shown in Supplementary Table 2. Main effects were observed in aSAT glucose transporter 4 (GLUT4), adiponectin, LPL, ATGL and TNFα mRNA (p < 0.05). In gSAT, main effects were further observed in adiponectin, and ATGL mRNA (p < 0.05). In only gSAT, NF-κB1 (p = 0.014) and TNFα (p = 0.039) gene expression were higher in the exercise than the control group at post-intervention. In both depots there was no change within or between groups for insulin receptor substrate (IRS)1, phosphoinositide 3-kinase (PI3K), peroxisome proliferator-activated receptors (PPAR)-γ, lipoprotein lipase (LPL), Diacylglycerol O-acyltransferase (DGAT) 2, perilipin 1 and catalase gene expression (p > 0.05).

### Baseline correlations of mitochondrial function with body fat distribution, ectopic fat and S_I_

**Figure 4:**
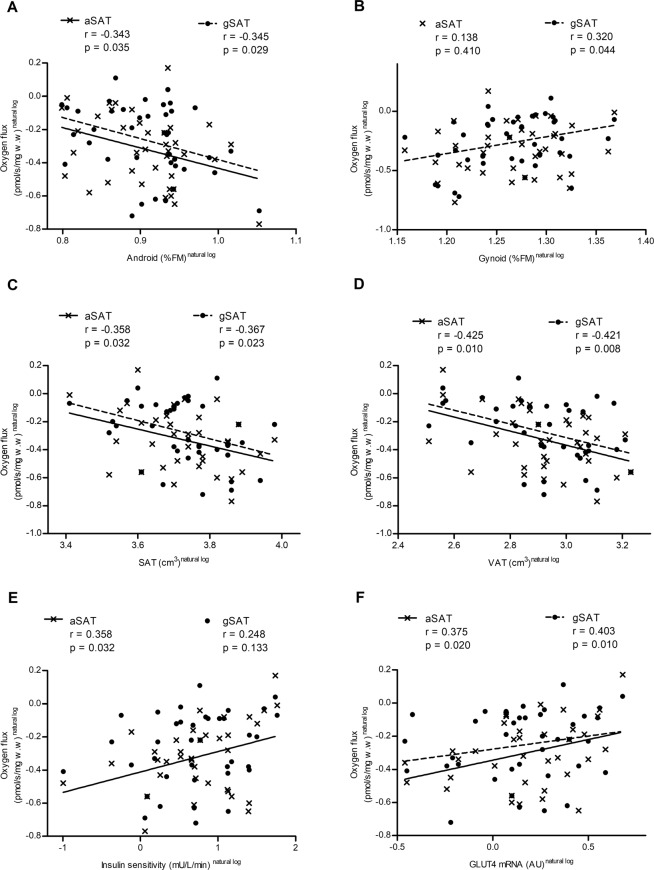
Baseline correlations on abdominal (aSAT) and gluteal (gSAT) subcutaneous adipose tissue mitochondrial oxygen flux, adjusted for mg w.w with body fat distribution (**A–D**), insulin sensitivity (**E**) and tissue specific gene expression of glucose transporter 4 (GLUT4) (**F**). All data not normally distributed and transformed prior to Pearson correlations. All data is pooled for graphical purposes and correlations were conducted on mitochondrial respiration in each depot. Sample numbers include, n = 38 (**A,B,F**) and n = 36 (**C,D,E**) in aSAT; n = 40 (**A,B,F**) and n = 38 (**C,D,E**) in gSAT. Mitochondrial oxygen flux represents Complex 1 and 2 linked respiratory state (oxidative phosphorylation capacity).

All correlations with mitochondrial function have been completed on the CI + II respiratory state as this represents the most physiologically relevant state of respiration and H_2_O_2_ production. Lower oxygen flux (mg.w.w-adjusted) in both aSAT and gSAT was associated with greater centralisation of body fat, characterised by higher android fat mass (%FM), abdominal VAT and SAT (p < 0.05; Fig. 4); however, these correlations were driven by mitochondrial content, with no association shown when adjusting mtDNA (Data not shown; p > 0.05). In contrast, lower oxygen flux (mg w.w-adjusted) in the gynoid depot was associated with higher gynoid fat mass (%FM; Fig. 4B), which remained significant when adjusting for mtDNA (Rho = 0.395, p = 0.031). Higher aSAT oxygen flux (mg.w.w-adjusted) was associated with higher S_I_ (Fig. 4E), which was no longer significant when adjusted for mtDNA (Rho = 0.101, p = 0.608). Higher oxygen flux (mg w.w-adjusted) in both depots were associated with greater depot-specific GLUT4 mRNA, however, when adjusted for mtDNA the association in remained significant in gSAT (Rho = 0.358, p = 0.052), but not aSAT (Rho = 0.253, p = 0.193).

Higher H_2_O_2_ production (mtDNA-adjusted) in gSAT was associated with lower total FM (kg) (Rho = −0.400, p = 0.026), and abdominal SAT volume (Rho = −0.425, p = 0.0191). These associations were no longer significant when adjusting for oxygen flux or mg w.w, which suggests the associations are driven by intrinsic mitochondrial function rather than content. Further, higher intrinsic mitochondrial H_2_O_2_ production in gSAT was associated with lower S_I_ (mtDNA-adjusted, Rho = −0.404, p = 0.024; Oxygen flux-adjusted, Rho = −0.430, p = 0.010), whereas higher H_2_O_2_ production in aSAT depot was associated with lower abdominal GLUT4 mRNA (mg w.w-adjusted, Rho = −0.384, p = 0.048; oxygen flux-adjusted Rho = −0.507, p = 0.002; mtDNA-adjusted, Rho = −0.384, p = 0.048). Mitochondrial function in both depots did not correlate with ectopic fat accumulation in all measured sites (p > 005; data not shown).

### Correlations of change in mitochondrial function with change in body fat distribution, ectopic fat and S_I_ in response to the intervention

**Figure 5:**
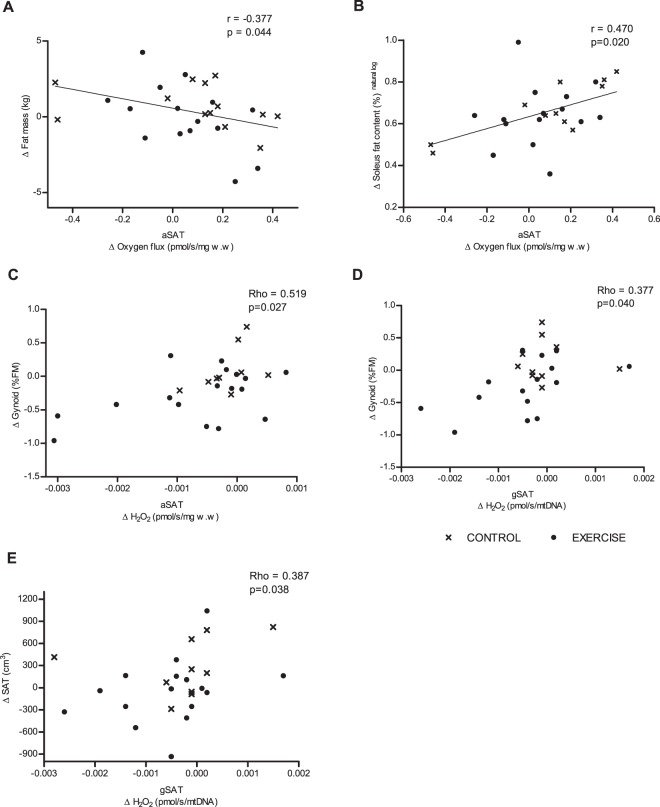
Correlations on change in abdominal subcutaneous adipose tissue (aSAT) mitochondrial oxygen flux, with change in fat mass (kg, **A**), soleus fat content (**B**). Change in H_2_O_2_ production in aSAT and gluteal SAT (gSAT) with change in gynoid fat mass (%FM) (**C,D**). Change in H_2_O_2_ production in gSAT with change in abdominal SAT volume (**E**). Data normally distributed and transformed (**A,B**) prior to Pearson correlations. Not normally distributed data are reported as Spearman’s correlations (**C,D,E**). All data is pooled, as no group interactions were evident. Sample numbers include, n = 29 (**A,D,E**), n = 26 (**B**) and n = 28 (**C**). Mitochondrial oxygen flux and H_2_O_2_ production represents complex 1 and 2 linked respiratory state (oxidative phosphorylation capacity).

There were no group x time interactions and all associations between changes in mitochondrial function and changes in body fat distribution and ectopic fat deposition are based on pooled data from exercise and control groups (Fig. 5). An increase in oxygen flux (mg w.w-adjusted) in aSAT correlated with an increase in hepatic fat (Rho = 0.430, p = 0.025) and soleus fat content (r = 0.470, p = 0.020; Fig. 5B), and a decrease in total body fat mass (r = −0.377, p = 0.044; Fig. 5A). These correlations were driven by mitochondrial content as they were no longer significant when adjust for change in mtDNA (p > 0.05; data not shown). Oxygen flux in aSAT and gSAT showed no other associations with body fat distribution, pancreatic fat content, S_I_ and GLUT4 mRNA (p > 0.05; data not shown). A decrease in aSAT H_2_O_2_ production (mg w.w-adjusted) correlated with a decrease in gynoid fat mass (%FM; Fig. 5C), which was no longer significant when adjusting for change in mitochondrial content (Rho = 0.277, p = 0.170). Conversely, a decrease in gSAT H_2_O_2_ production (mtDNA-adjusted) correlated with decrease in gynoid fat mass (FM%) and abdominal SAT volume (p < 0.05; Fig. 5D,E), which were determined by intrinsic mitochondrial characteristics rather than content. H_2_O_2_ production (all adjustments) showed no associations with S_I_ in either aSAT or gSAT (p > 0.05; data not shown).

## Discussion

This is the first human study to show unique depot-specific characteristics in SAT mitochondrial function that were related to body fat distribution and S_I_. When compared to aSAT, gSAT has a higher mitochondrial respiration capacity and a higher production of H_2_O_2_, which correlated with higher gynoid fat mass and lower S_I_, respectively. In comparison, the lower mitochondrial respiration in aSAT was related to a higher central fat distribution (VAT and SAT) and lower S_I_. In response to exercise training, mitochondrial respiration and coupling increased in aSAT, while H_2_O_2_ production decreased in gSAT. These adaptations in mitochondrial function occurred alongside improvements in S_I_, and a small reduction in gynoid fat mass. Interestingly, when investigating the change in mitochondrial function in response to the exercise intervention, there was no relationships with S_I_ in either depot, but rather the increase in aSAT mitochondrial respiration correlated with a decrease in total fat mass and an increase in hepatic and soleus fat content. The outcomes to this study highlight the importance of understanding mitochondrial function in the gluteal and abdominal SAT depots when investigating the pathophysiology of insulin resistance and associated risk factors such as body fat distribution and ectopic lipid deposition. Furthermore, we highlight the benefits of exercise training in stimulating positive adaptations in mitochondrial function in multiple SAT depots.

Intrinsic mitochondrial function in adipocytes is dependent on mitochondrial number and/or capacity^11^. We showed that gSAT has higher mitochondrial ETS capacity compared to aSAT; but this was not reflected in differences in mitochondrial coupling (respiratory control ratios) or content. Given there were no differences in mitochondrial content between depots, the higher ETS capacity in the gSAT may be required to ensure the storage capacity of the cell^1^. Our results show a higher CI + II linked respiration in gSAT is associated with higher gynoid fat mass (%FM), and lower central fat accumulation (abdominal SAT and VAT volume). Mitochondrial respiration in aSAT was the only depot to show a correlation with lower S_I_. Interestingly, associations with abdominal fat distribution and S_I_ were no longer significant when adjusting for mtDNA, which suggested they were driven by mitochondrial content rather than intrinsic function. These results collectively suggest that mitochondrial respiration in gSAT specifically relates to regional body fat distribution, while mitochondrial respiration in aSAT relate to abdominal fat distribution and S_I_. In support of this, a study using a mouse model showed that subcutaneous flank fat transplantation into the abdominal cavity (visceral and subcutaneous depots) improved whole-body glucose homeostasis^3^, while increased insulin sensitivity in participants with type 2 diabetes treated with Pioglitazone administration increased the expression of genes related to mitochondrial biogenesis in aSAT^21^. Although our results do not show cause and effect, they do suggest a direct relationship between mitochondrial respiration in aSAT and whole-body S_I_, which requires further exploration.

Obese black South African women with normal glucose tolerance have been shown to favour gynoid fat distribution^9^, and present with adipose tissue hypertrophy of the gluteal region^1,22^. Consistent with these findings, the participants in our study presented with a relatively low waist-to-hip ratio (mean ± 0.89) despite being obese. Notably, the higher H_2_O_2_ emissions in the gluteal tissue at baseline may signify cellular stress related to higher SOD activity and an over flux of fatty acids into the mitochondria and causing, I) increased fat oxidation and subsequent ROS production, which may explain the higher ETS capacity in gluteal SAT, and II) incomplete β-oxidation triggering a build-up of acetyl CoA to be diverted to triglyceride synthesis and storage^23^. Our results also show that higher gSAT H_2_O_2_ emissions are related to lower S_I_ and lower abdominal SAT volume. We hypothesise that the gynoid depot is able to store excess fatty acids, which may protect against fatty acid deposition in abdominal SAT but result in increased mitochondrial stress that associates with decreased S_I_. Although gynoid fat is considered to be a depot that protects against the development of insulin resistance^4^, our results suggest that there may be a point during obesity where the gynoid fat starts to contribute to the pathology of type 2 diabetes. Indeed, we have previously shown that the gluteal depot of obese black South African women is associated with higher expression of genes related to hypoxia and collagen deposition, which associated with reduced S_I_^24^.

In addition to mitochondrial function, inflammation and ROS production in adipose tissue are mechanisms of cellular stress that can initiate lipolysis and the redistribution of fatty acids to visceral and ectopic depots. Previous research suggests a positive feedback loop between TNF-α and ROS, with catalase being important to counteract the TNF-α induced apoptosis by neutralizing mitochondrial ROS^13^. There were no differences in inflammatory or catalase gene expression between depots, which presents a disconnect between inflammatory gene expression and mitochondrial H_2_O_2_ production in gSAT. This disconnect may be related to cytoplasmic ROS during early stages of obesity and/or changes in other aspects of oxidative stress (i.e. 4-HNE, protein carbonylation) or the antioxidant system (i.e. Glutathione peroxidase, SOD activity). However, this was not analysed in the present study and is an avenue for future research^23^. Furthermore, it is not unreasonable to suggest that the higher lipolytic profile (ATGL gene expression) in gSAT may be a response to the higher mitochondrial H_2_O_2_ production at baseline and the small reduction in gynoid fat mass (%FM) in response to 12 weeks of exercise training. In response to the exercise training gSAT was the only depot that showed the most consistent and significant reduction in H_2_O_2_ emissions across multiple respiratory states. These changes in mitochondrial H_2_O_2_ emissions and content in gSAT were associated with the reduction in gynoid fat mass. Furthermore, these changes occurred without changes in catalase gene expression, but a higher inflammatory profile (TNF-α and NF-κB gene expression) post-exercise training, which may reflect tissue remodelling. Collectively, mitochondrial H_2_O_2_ production in gSAT may be an indicator of higher SOD activity and the aforementioned cellular stress from the over flux of fatty acids into the mitochondria and subsequent excess triglyceride storage during obesity, and exercise training is a practical stimulus for ameliorating these cellular stressors.

This study presents unique data on the different mitochondrial respiration adaptations in aSAT and gSAT in response to exercise training. In response to 12-weeks of exercise training, aSAT, but not gSAT, increased in intrinsic mitochondrial CI and CI + II linked respiration, and ETS capacity. Specifically, increased aSAT CI + II linked respiration correlated with a decrease in total body fat mass. These findings complement previous literature that shows mitochondrial oxidative capacity in aSAT is lower in obese compared with non-obese adults and is related to overall adiposity rather than adipocyte hypertrophy^25^. Although the present study does not show that exercise training significantly changed abdominal fat content or ectopic lipid deposition, the increase in aSAT mitochondrial respiration and coupling in response exercise training may suggest the early stages of tissue remodelling. Furthermore, the increase in intrinsic mitochondrial respiration in only aSAT in response to exercise training was associated with an increase in soleus muscle and hepatic fat content. However, when accounting for the change in mitochondrial content, these associations were no longer significant. Accordingly, mitochondrial content in aSAT may play a role in improved metabolic flexibility and the subsequent redistribution of fatty acids towards the liver and skeletal muscle for beta-oxidation^12^. A previous study showed that the addition of exercise training to a Palaeolithic diet resulted in a heterogeneous response of liver and muscle fat deposition, despite improved metabolic health. The authors concluded that lipid storage in the liver may be as dynamic as lipid storage in the skeletal muscle in response to lifestyle interventions^26^. Collectively, these results demonstrate that adaptations in mitochondrial function in aSAT may precede changes in abdominal fat and ectopic lipid deposition and a longer training program may be required to obtain more substantial adaptations.

At baseline, a higher CI + II linked respiration in both depots was associated with increased GLUT4 expression, which is known to facilitate lipogenesis. Despite the positive baseline correlations between aSAT mitochondrial respiration and S_I_, the increase in S_I_ in response to exercise training was not directly related to increased aSAT respiration or GLUT4 gene expression, albeit they improved simultaneously. This may suggest that the nature of the relationship between aSAT mitochondrial function and S_I_ is altered and mediating factors such as lipid metabolism and/or mitochondrial function in skeletal muscle may be involved. In an overweight cohort (80% men), 6 weeks of high-intensity exercise training did not change mitochondrial oxidative phosphorylation capacity in aSAT, but rather resulted in increased mitochondrial function in skeletal muscle^27^. Considering there were no increases in lipogenic (LPL, Perilipin 1, DGAT2, IRS1 and PI3K), lipolytic (ATGL) and insulin signalling (IRS1 and PI3K) genes, the increase in GLUT4 mRNA and mitochondrial respiration in the exercise group suggests an increase in AMPK-mediated glucose entry and metabolism into aSAT^28^. Although AMPK was not measured in the current study, it may explain the increase in GLUT4 in response to training, independent to the insulin signalling cascade. In rodent models, exercise training has been shown to increase the enzymes associated with mitochondrial biogenesis, oxygen consumption rate, GLUT4 and reduce UCP2 in SAT (inguinal)^29,30^. Similarly, aSAT was the only depot in which oxygen flux through Leak^ETF^ respiration was reduced and mitochondrial coupling control ratios increased, which was further supported our increase in GLUT4 gene expression in response to exercise training. Taken together, the increase in abdominal GLUT4 mRNA occurred alongsideincreased mitochondrial respiration and coupling, which strongly suggests that exercise training stimulated aSAT to be a more metabolically active tissue compared to gSAT.

An unexpected finding was the reduction in mitochondrial content in gSAT in response to exercise training, which suggests less mitochondria for an equivalent functional capacity. MtDNA is a common marker for mitochondrial content in adipose tissue^2,27^, however, no studies have compared the accuracy of mtDNA with other markers used in skeletal muscle, such as citrate synthase activity and content, OXPHOS content and transmission electron microscopy^31^. The mitochondria are a continuing source of ROS production, which can accumulate and impair the synthesis of key proteins for oxidative phosphorylation^32^. We hypothesise that high baseline mitochondrial H_2_O_2_ emissions in the gSAT signal mitophagy in response to exercise training, which is a key mechanism for the selective elimination of dysfunctional mitochondria^32^. This reduction in mitochondrial content may be a necessary short-term training adaptation to ensure more long-term improvements in mitochondrial function and coupling. Mitophagy and its associated mediators (i.e. AMPK signaling) in response to exercise training has only been explored in human skeletal muscle, and further research is required to explore this across adipose tissue depots.

Of note, the control group showed significant increases in weight and abdominal SAT volume, which is consistent with previous literature reporting that young (age 27 ± 8 years) obese free-living black South African women show a consistent increase in weight (6.9 ± 9.9 kg) and central fat accumulation over a 5.5 year period^33^. The changes in body composition in the control group coincide with a decrease in aSAT and gSAT adiponectin gene expression. Adiponectin is an insulin sensitising hormone produced by adipocytes and the reduction may suggest an increase in insulin resistance in both SAT depots of the control group^34^. Moreover, in the aSAT depot of the control group, there was also an increase in GLUT4 and TNF-α gene expression, and intrinsic CI + II linked respiration. These changes may be an adaptation to weight gain that reflects a chronic excess supply of substrates and storage of fatty acids, whilst highlighting the importance of including a measure of tissue function or activity (i.e. mitochondrial function) when understanding changes in gene expression. These results suggest a potential role of SAT mitochondrial function in overall weight gain and abdominal SAT accumulation in the control group and highlights the protective role of exercise training in obese populations.

Whilst this study provides novel insight into depot-specific adipose tissue mitochondrial function, there are limitations that need to be addressed when interpreting these data. Firstly, the cohort of interest are obese, black South African women, who present with a unique phenotype of low centralised fat and a high insulin resistance. Further research is required in all women (obese, overweight and normal weight) that exhibit a phenotype favouring abdominal fat accumulation. Moreover, differences in depot-specific adipose tissue mitochondrial function between genders are also unknown and requires further investigation. This will provide further insight into the contribution of mitochondrial function to body fat distribution and associated risk for the development of type 2 diabetes. Although this study provides novel information on mitochondrial function across depots, the study is invasive and accordingly the samples size is small and limits the extrapolation of results to the general population. Furthermore, a small sample volume of adipose tissue was collected, thus protein expression of the investigated genes was not quantified. In addition, future research should focus post-translation modifications in response to exercise training and the association with adaptations in mitochondrial functioning. Furthermore, the normalization of wet weight tissues relies on the modulation of cell size and density, which would be expected to change, both in response to exercise training and weight gain shown in the control group. However, cell size and density were not measured in the current study and results need to be interpreted accordingly.

The results of this study highlight novel differences in mitochondrial function between SAT depots at baseline and in response to 12-weeks of exercise training in obese black South African women. Notably, the improved mitochondrial coupling in aSAT and reduced H_2_O_2_ production in gSAT in response to exercise training infers potential depot-specific targets for reducing fat mass and increasing S_I_. Finally, we suggest that mitochondrial function in SAT may be target mechanisms for understanding the pathophysiology insulin resistance and type 2 diabetes in an obese cohort, with a view of developing targeted treatment and prevention strategies.

## Methods

### Study design

This randomized controlled research study recruited 45 obese sedentary black South African women, who were block (2–4 participants) randomized into control (n = 22) or experimental (exercise, n = 23) groups. Group allocation occurred after pre-intervention testing. Accordingly, investigators were blinded to the group allocation during pre-intervention testing, however, investigators were not blinded during post-intervention testing. Detailed information regarding recruitment, retention, analyses (CONSORT) and methods are reported in^20^. Ten participants dropped out of the study or were lost to follow-up. The exercise (n = 20) completed 12 weeks of supervised combined aerobic and resistance training. Both the control group (n = 15) and exercise groups were instructed to continue their habitual physical activity and dietary behaviors. This study was approved by the Human Research Ethics Committee at the University of Cape Town (HREC REF: 054/2015) and registered in the Pan African Clinical Trial Registry (PACTR201711002789113). The study was performed in accordance with the principles of the Declaration of Helsinki (1964, amended last in Fortaleza Brazil, 2013), ICH Good Clinical Practice (GCP), and the laws of South Africa. Participants provided written informed consent before screening and participation.

### Participants

Participant recruitment ensured the following inclusion criteria: black South African women (based on the *isiXhosa* ancestry of both parents), 20–35 years of age, body mass index (BMI) of 30–40 kg/m^2^, weight stable (weight not changed more than 5 kg or no change in clothes size over the past 6 months), sedentary (not participating in exercise training; >1 session of >20 min per week), on injectable contraceptive (depot medroxyprogesterone acetate, 400 mg) for a minimum of 2 months, no known metabolic or inflammatory diseases, no hypertension (≥140/90 mmHg; Omron 711, Omron Health Care, Hamburg, Germany), no diabetes [random plasma glucose concentration of >11.1 mmoL/L, and/or haemoglobin A_1c_ (HbA_1c_) >6.5%], HIV negative (rapid HIV screening test kit), no anaemia (haemoglobin (Hb) <12 g/dL), not taking any medications, non-smokers, no orthopaedic or medical problems that may prevent exercise participation, and no surgical procedures within the last 6 months.

### 12-week intervention

The exercise intervention consisted of 12-weeks of supervised aerobic and resistance training at a moderate-vigorous intensity for 40–60 min, four days per week by a trained facilitator. Aerobic exercises included dancing, running, skipping, and stepping at a moderate-vigorous intensity (75–80% peak heart rate; HR_peak_). Resistance exercises at a prescribed intensity of 60–70% HR_peak_, included upper and lower-body exercises at body weight that progressed to the use of equipment (i.e. bands and free weights). A heart rate monitor (Polar A300, Kempele, Finland) was worn to ensure the prescribed exercise intensity was maintained. Both groups were instructed to maintain their usual dietary intake and physical activity patterns, which was objectively quantified at baseline, weeks 4, 8 and 12. Following post-intervention testing, the control participants were provided with the opportunity to participate in the 12-week supervised exercise program.

### Pre- and Post-intervention Testing

#### Body composition assessment

Basic anthropometry, including weight, height, and waist (level of umbilicus) and hip circumference (largest protrusion of the buttocks), were measured to the nearest 0.1 cm. Whole body composition, including subtotal (excluding the head) fat mass and fat-free soft tissue mass (FFSTM), were measured by dual-energy X-ray absorptiometry (DXA; Discovery-W, software version 12.7.3.7; Hologic, Bedford, MA) according to standard procedures. Regional body fat distribution, including gynoid and android fat mass was characterized as previously described^35^.

#### Cardiorespiratory fitness

A walking, treadmill-based (C, Quasar LE500CE, HP Cosmos, Nussdorf-Traunstein, Germany) graded exercise test determined peak oxygen consumption (VO_2peak_) and peak heart rate (HR_peak_; Polar A300, Kempele, Finland). Pulmonary gas exchange was measured by determining O_2_ and CO_2_ concentrations and ventilation to calculate VO_2_ consumption using a metabolic gas analysis system (CPET, Cosmed, Rome Italy). A 2-point calibration was conducted prior to each test, as previously described^20^.

#### Frequently sampled intravenous glucose tolerance test (FSIGT)

Baseline samples were collected at −5 and −1 min before a bolus of glucose (50% dextrose; 11.4 g/m^2^ × body surface area) was infused intravenously over 60 s beginning at time 0. At 20 min, human insulin (0.02 U/kg; NovoRapid, Novo Nordisk) was infused over 5 min at a constant rate (HK400 Hawkmed Syringe Pump, Shenzhen Hawk Medical Instrument Co., Shenzhen, China) and samples were collected up to 240 min. Bergman’s minimal model of glucose kinetics was used to calculate the insulin sensitivity index (S_I_)^36^. Samples for insulin (IMMULITE 1000 immunoassay system, Siemens Healthcare, Midrand, South Africa) and glucose (Randox, Gauteng, South Africa) were collected in serum-separating and fluoride oxalate tubes, respectively. Samples were centrifuged at 3000 rpm for 10 min at 4 °C and stored at −80 °C until further analyses.

#### Ectopic lipid content

After a standardized meal (Energy: 2553 kJ), MRI was used to determine hepatic, pancreatic and skeletal muscle (soleus, tibialis anterior) fat content using a 3 Tesla whole-body human MRI scanner (MAGNETOM Skyra, Siemens Medical Solutions, Erlangen, Germany) using previously described techniques. Region of interests (ROI) were manually drawn, using OsiriX software, on 7 consecutives slices in both the right lobe of the liver and in the soleus and tibialis anterior muscles of the calf^37^. A total of 3 circular (1 cm^2^) ROIs were drawn in the pancreas; one ROI located in the head, body and tail^38^. The fat fraction was calculated as the fat signal over the sum of the water and fat signals Abdominal VAT and SAT volumes were determined by calculating the sum of the VAT and SAT areas from 5 images in a 15 cm region from the level of L1–5 and then multiplied by 3^38^.

#### Adipose tissue biopsies

After a 4–6 h fast and at least 48–72 h after the last exercise session, fat samples were collected. Fat samples were obtained using a mini-liposuction technique^1,20^. Abdominal samples were from directly above the umbilicus, and gluteal samples were from the right upper outer quadrant. Samples were washed with normal saline until no blood was visible. A subsample was placed in ice-cold BIOPS^25^ for immediate analysis of mitochondrial respiration and H_2_O_2_ emissions. The remaining samples were frozen immediately in liquid nitrogen (N_2_) and stored at −80 °C for the analysis of mitochondrial DNA (mtDNA), gene expression and protein content.

#### Adipose tissue gene expression and mitochondrial DNA (mtDNA)

RNA was extracted from gSAT and aSAT using the RNeasy Mini lipid kit (Qiagen Ltd, Germantown, MD, USA). The concentration and purity were determined spectrophotometrically using a microplate data acquisition program (Synergy HT, Gen5 2.01; Biotek Instruments, Inc; Vermont, USA) and the RNA integrity was analysed by 1% agarose gel electrophoresis. RNA was reverse transcribed to cDNA using the High-Capacity cDNA Reverse Transcription Kit with RNase inhibitors (Thermofisher Scientific, Waltham MA, USA). Real-time PCR (RT-PCR) was performed in triplicate using Applied Biosystems QuantStudio^TM^ 3 RT-PCR system with predesigned Taqman assays (Thermofisher Scientific, Waltham MA, USA). Gene expression included, Insulin receptor substrate 1 (IRS1; Hs00178563_m1), Phosphoinositide 3-Kinase (PI3K; Hs00979691_m1), Glucose transporter 4 (GLUT4; Hs00168966_m1), Peroxisome proliferator-activated receptors (PPARγ; Hs01115513_m1), adiponectin (Hs00605917_m1), Adipose triglyceride lipase (ATGL; Hs00386101_m1), Lipoprotein lipase (LPL; Hs00173425_m1), Perilipin 1 (Hs00160173_m1), TNFα (Hs00174128_m1), NFκB-p105 (Hs00765730_m1) and catalase (Hs00156308_m1). A standard curve was constructed for each primer probe set using a serial dilution of cDNA pooled from all samples. All gene are presented as the ratio of abundance of the gene of interest: mean of abundance of the relevant endogenous gene (RPLPo; Hs99999902_m1).

Genomic DNA was extracted from 80–100 mg of adipose tissue using the DNeasy Blood and Tissue Handbook mini kit, according to the manufacturer’s instructions (Qiagen, Hilden, Germany). DNA concentrations and purity were quantified using the Nanodrop spectrophotometer (Nanodrop Technologies, Wilmington DE, USA). RT-PCR was conducted using 15 ng of DNA, and the nuclear -globin (Hs00758889_s1) and mitochondrial ND2 gene (Hs02596874_g1) TaqMan Gene Expression Assays and universal master mix using the QuantStudio^TM^ 7 Real-Time PCR system according to the manufacturer’s instructions (Thermo Fisher Scientific, Waltham MA, USA). A standard curve was constructed for each primer probe using a serial dilution of pooled DNA from all samples. Mitochondrial DNA was determined relative to the nuclear-globin gene using the standard curve method of quantification. Results reflected the number of mtDNA molecules that existed for each nDNA molecule and since there are two copies of nDNA within each human cell, this answer was then multiplied by two^39^.

#### Adipose Tissue Mitochondrial Respiratory Function

Mitochondrial function was defined as measured respiration and H_2_O_2_ production that were performed in respiration medium (MiR05) at 37 °C using the high-resolution Oxygraph-2k (Oroboros, Innsbruck, Austria). All measures were completed in duplicate and carried out in a hyper-oxygenated (250–450 nmoL/mL) environment. Adipose tissue samples were prepared and analyzed according to previously described methods^2,40^. Briefly, immediately after tissue collection, samples were stored in ice-cold preservation solution (BIOPS)^40^ for a maximum of 4 hours before analyses. Adipose tissue (50–60 mg w.w) were permeabilized in saponin (5 mg/mL; Saponin/BIOPS) for 20 min and washed in MiR05 for 2 × 10 min. The multiple SUIT protocol included^27,41^: (1) Electron flow through electron transferring flavoprotein (ETF) and medium chain fatty-acid oxidation in the absence of adenylates (Leak^ETF^) with the addition of malate (2 mM) and octanoyl-carnitine (0.2 mM); (2) Maximal flow of electrons through ETF and fatty-acid oxidation (ETF^p^) with the addition of ADP (5 mM); (3) State 3 respiration capacity (CI) specific to ETF and complex I (pyruvate 5 mM; glutamate 10 mM); (4) Maximal state 3 respiration (CI + II), oxidative phosphorylation capacity (Succinate, 10 mM); (5) State 4o respiration, oligomycin-induced leak respiration (Leak^oly^) through inhibition of ATP synthase (Oligomycin 2.5 μM); (6) Electron transport system (ETS) capacity with the titration of CCCP (0.5 μM titration steps); (7) Inhibition of complex I with the addition of rotenone (0.5 μM); (8) The inhibition of complex III with the addition of antimycin A (2.5 μM). Complex III inhibition was used for the determination and correction of residual oxygen consumption (non-mitochondrial oxygen consumption in the chamber).

Hydrogen peroxide (H_2_O_2_) flux was measured simultaneously with respirometry in the O2k-Fluorometer (O2k-Fluo LED2-Module Fluorescence-Sensor Green) using the H_2_O_2_ sensitive probe Amplex UltraRed. Amplex UltraRed (10 μM) and 1 U/mL horseradish peroxidase (HRP) was added to the chamber. Amplex red reacts with H_2_O_2_ in the presence of HRP producing resorufin, which can be measured fluorometrically. Calibrations were performed throughout the respirometry experiment to account for degradation of fluorescence over time, with 2 steps of H_2_O_2_ additions of 0.1 μM per step. This protocol relies on endogenous SOD production to neutralise superoxide and produce H_2_O_2._ Mass-specific H_2_O_2_ (mg w.w) were calculated relative to mtDNA (H_2_O_2_/mtDNA) and oxygen flux (H_2_O_2_/*J*O_2_)^42^. Notably, mtDNA was divided into mass-specific H_2_O_2_ to calculate (H_2_O_2_/mtDNA) and oxygen flux was divided into mass-specific H_2_O_2_ to calculate (H_2_O_2_/*J*O_2_).

### Monthly monitoring

#### Physical activity and dietary intake

Physical activity was measured using accelerometery (ActiGraph GTX3 +, ActiGraph LLC, Pensacola FL, USA), at baseline, weeks 4, 8, and 12. The ActiGraph was worn on the right hip for 24 hours a day over a 7-day period. Physical activity was analyzed using the ActiLife Software Version 6 (ActiLife LLC). At the same time points dietary intake was estimated using a 24-hour recall and a 3-day dietary record, which included 2 weekdays and 1 weekend day. Nutrient intake was calculated using the South African Food Composition Database System (SAFOOD, the South African Food Composition Database, South African Medical Research Council, Cape Town, South Africa).

#### Statistics

Data was analysed using IBM SPSS statistics (Version 25, Statistical Package for the Social Sciences, Chicago, IL, USA). Normally distributed data are expressed as mean ± standard deviation (SD) and non-normally distributed data are expressed as median (interquartile range; IQR), and transformed prior to analysis. Paired t-tests were used for baseline comparisons and mixed-model analyses with main effects of time (pre and post intervention), group (exercise and control) and interaction effects (group x time). When significant main effect or interaction a Fisher’s Least Significant Difference post-hoc test was used. Statistical significance (alpha) was set at p < 0.05. Spearman’s correlations were conducted on baseline data (n = 45) and on the change in response to the intervention (exercise, n = 20; control n = 15; n = 35 pooled data set).

## Supplementary information

Supplementary tables and figures.
