# Supplementary material for: Exercise training results in depot-specific adaptations to adipose tissue mitochondrial function
Source: Sci Rep. 2020 Mar 2;10:3785. doi: 10.1038/s41598-020-60286-x (PMC7052157; doi:10.1038/s41598-020-60286-x)
Supplement: Supplementary file 1 — Supplementary tables and figures. [file 41598_2020_60286_MOESM1_ESM.pdf]

**Article Title**

Exercise training results in depot-specific adaptations to adipose tissue mitochondrial function.

**Authors**

Mendham, Amy E., Larsen, Steen., George, Cindy., Adams, Kevin., Hauksson, Jon., Olsson, Tommy., Fortuin-de Smidt, Melony C., Nono Nankam, Pamela, Hakim, Olah, Goff, Louise M, Pheiffer, Carmen., Goedecke, Julia H

**Supplementary table 1: Energy expenditure and consumption at baseline, weeks 4, 8 and 12 of the intervention.**

| Variable | EXERCISE | | | | CONTROL | | | | Time | Group | Interaction |
|---|---|---|---|---|---|---|---|---|---|---|---|
| | Baseline | Week 4 | Week 8 | Week 12 | Baseline | Week 4 | Week 8 | Week 12 | P Value | P Value | P Value |
| Energy expenditure (kJ/day)[1] | 2426 (2266,3025) | 2160 (1734,2940) | 2640 ± 959 | 2736 ± 792 | 2501 (2346,3149) | 2831 (2400,3544) | 2916 ± 964 | 2837 ± 966 | 0.722 | 0.335 | 0.285 |
| Metabolic Equivalents (h/day) | 1.15 (1.11,1.18) | 1.10 (1.08,1.20) | 1.15 ± 0.08 | 1.15 ± 0.05 | 1.13 (1.09,1.17) | 1.16 (1.09,1.21) | 1.15 ± 0.05 | 1.14 ± 0.06 | 0.544 | 0.975 | 0.869 |
| Energy Intake (kJ/day) | 8369 (6984,11079) | 8132 ± 2042 | 8765 ± 2178 | 8615 ± 1668 | 8138 (6493,9434) | 7883 ± 1338 | 7880 ± 1857 | 8661 ± 1354 | 0.267 | 0.434 | 0.632 |
| Carbohydrates (%EI) | 55.1 ± 5.5 | 53.9 ± 5.9 | 54.2 ± 5.3 | 52.7 ± 8.3 | 54 ± 5.7 | 56.9 ± 6.0 | 56.0 ± 7.6 | 57.8 ± 7.2 | 0.913 | 0.202 | 0.322 |
| Fats (%EI) | 30.4 ± 6.1 | 31.0 ± 6.0 | 31.3 ± 5.5 | 32.2 ± 5.9 | 31.0 ± 5.6 | 29.2 ± 5.1 | 30.0 ± 7.3 | 27.9 ± 6.4 | 0.970 | 0.485 | 0.235 |
| Protein (%EI) | 13.2 ± 2.5 | 13.9 ± 2.9 | 13.4 ± 2.0 | 13.7 ± 3.0 | 14.3 ± 1.9 | 13.2 ± 2.3 | 13.8 ± 2.8 | 13.6 ± 2.1 | 0.995 | 0.845 | 0.575 |

Normally distributed data reported as Mean ± Standard Deviation and skewed data reported as Median (Interquartile Range; IQR). Repeated-measures ANOVA identified main effects of time (pre and post) and group (exercise and control), and interactions (group x time) effect for exercise (n=20) and control (n=15) groups. Physical activity on non-exercise training days in the exercise group[1]. EI, Energy Intake.

**Supplementary Table 2: Adipose tissue gene expression that represent mitochondrial content, insulin signalling, lipolysis, lipogenesis and inflammation pre and post 12-week intervention.**

| Variable | BASELINE | EXERCISE | | CONTROL | | Time | Group | Interaction |
|---|---|---|---|---|---|---|---|---|
| | | Pre | Post | Pre | Post | P Value | P Value | P Value |
| ***Abdominal Subcutaneous Adipose Tissue*** | | | | | | | | |
| *Mitochondrial content* | | | | | | | | |
| Mitochondrial DNA (AU) | 2.25 (1.92,3.18) | 2.5 (1.9,3.2) | 2.1 (1.5,2.7) | 2.0 (1.6,3.0) | 1.9 (1.8,2.5) | 0.211 | 0.544 | 0.247 |
| *Insulin Signalling* | | | | | | | | |
| Glut4 mRNA (AU) | 1.39 (1.00,2.14) | 1.39 (0.61,2.82) | 1.73 (1.24,2.25) | 1.18 (1.01,1.64) | 1.90 (1.15,3.17) | **0.002** | 0.677 | 0.990 |
| IRS1 mRNA (AU) | 1.32 (0.95,2.10) | 1.40 (0.91, 2.10) | 1.28 (0.90,1.98) | 1.11 (0.85,1.78) | 1.30 (0.82,1.76) | 0.918 | 0.365 | 0.113 |
| PI3K mRNA (AU) | 1.43 ± 0.51 | 1.28 (1.03,1.66) | 1.20 (1.00,1.87) | 1.35 (1.05,1.66) | 1.20 (0.72,1.72) | 0.918 | 0.365 | 0.113 |
| Adiponectin mRNA (AU) | 0.78 ± 0.33 | 0.76 (0.43,0.93) | 0.62 (0.40, 0.88) | 0.78 (0.52,0.98) | 0.53 (0.48,0.69) | **0.016** | 0.650 | 0.611 |
| PPAR-γ mRNA (AU) | 1.06 ± 0.46 | 1.14 (0.78,1.39) | 0.85 (0.55,1.06) | 0.90 (0.62,1.37) | 0.78 (0.63,1.30) | 0.073 | 0.945 | 0.297 |
| *Lipolysis* | | | | | | | | |
| ATGL mRNA (AU) | 1.22 ± 0.53 | 0.98 (0.76,1.72) | 0.95 (0.61,1.23) | 1.45 (0.97,1.78) | 1.08 (0.78,1.38) | 0.069 | 0.486 | 0.239 |
| *Lipogenesis* | | | | | | | | |
| LPL mRNA (AU) | 1.01 ± 0.40 | 0.94 (0.79,1.21) | 0.74 (0.55,1.10) | 1.07 (0.75,1.26) | 0.75 (0.61,0.89) | **0.035** | 0.592 | 0.654 |
| DGAT2 mRNA (AU) | 0.93 (0.64,1.77) | 0.73 (0.24,2.17) | 1.59 (0.94,3.00) | 0.93 (0.71,1.89) | 1.57 (0.83,2.39) | 0.076 | 0.997 | 0.632 |
| Perilipin 1 mRNA (AU) | 2.00 (1.16,3.10) | 1.69 (1.01,2.47) | 1.56 (1.22,2.57) | 2.22 (1.59,3.08) | 1.57 (1.14,2.38) | 0.259 | 0.527 | 0.373 |
| *Inflammation* | | | | | | | | |
| NF-κB1 mRNA (AU) | 1.36 (1.08,2.1) | 1.23 (1.01,2.15) | 1.51 (1.01,2.04) | 1.32 (0.96,1.63) | 1.04 (0.90,1.59) | 0.957 | 0.537 | 0.196 |
| TNF-α mRNA (AU) | 0.90 (0.55,1.32) | 0.93 (0.54,1.31) | 1.33 (0.65,1.72) | 0.72 (0.54,1.03) | 0.95 (0.75,1.43) | **0.004** | 0.722 | 0.992 |
| Catalase mRNA (AU) | 1.77 ± 0.58 | 1.67 ± 0.59 | 1.61 ± 0.56 | 1.63 ± 0.54 | 1.66 ± 0.36 | 0.226 | 0.335 | 0.626 |
| ***Gluteal Subcutaneous Adipose Tissue*** | | | | | | | | |
| *Mitochondrial content* | | | | | | | | |
| Mitochondrial DNA (AU) | 2.19 ± 0.45 | 2.4 (2.2,2.6) | 2.2 (1.6,2.4) | 2.2 (1.9,2.9) | 2.0 (1.9,2.5) | **0.031** | 0.749 | 0.719 |
| *Insulin Signalling* | | | | | | | | |
| Glut4 mRNA (AU) | 1.43 (0.99,1.98) | 1.41 (0.89,2.07) | 1.69 (1.19,2.27) | 1.38 (1.17,1.71) | 1.62 (0.79,2.85) | 0.265 | 0.966 | 0.521 |
| IRS1 mRNA (AU) | 1.73 (1.36,2.19) | 1.24 (0.91,1.53) | 1.28 (1.05,1.56) | 1.08(0.92,1.62) | 1.12 (0.94,1.85) | 0.705 | 0.569 | 0.626 |
| PI3K mRNA (AU) | 1.38 (1.11,1.79) | 1.32 (1.09,1.53) | 1.42 (1.19,1.86) | 1.49 (1.11,1.81) | 1.15 (1.00,1.92) | 0.892 | 0.215 | 0.735 |
| Adiponectin mRNA (AU) | 0.69 (0.52,0.96) | 0.68 (0.56,0.77) | 0.64 (0.50,0.83) | 0.84(0.50,1.09) | 0.52 (0.47,0.66) | **0.029** | 0.628 | 0.161 |
| PPAR-γ mRNA (AU) | 1.12 ± 0.43 | 1.03 (0.84,1.39) | 0.98 (0.84,1.22) | 1.20 (0.81,1.35) | 0.82 (0.64, 1.46) | 0.276 | 0.720 | 0.407 |
| *Lipolysis* | | | | | | | | |
| ATGL mRNA (AU) | 1.35 (1.09,2.19)^ | 1.39 (1.05,1.70) | 1.17 (0.86,1.48) | 1.53 (1.00,2.55) | 1.19 (0.91,1.49) | **0.003** | 0.630 | 0.393 |
| *Lipogenesis* | | | | | | | | |
| LPL mRNA (AU) | 1.02 (0.73,1.39) | 1.03 (0.81,1.33) | 0.81 (0.67,1.15) | 1.34 (0.82, 1.51) | 1.06 (1.38,0.70) | 0.071 | 0.455 | 0.654 |
| DGAT2 mRNA (AU) | 1.35 (0.83,1.79) | 1.29 (0.72,1,92) | 1.23 (1.02,1.94) | 1.53 (0.99,2.39) | 1.23 (0.59,2.29) | 0.531 | 0.739 | 0.802 |
| Perilipin 1 mRNA (AU) | 1.97 ± 0.65 | 1.76 (1.44,2.39) | 1.17 (0.86,1.48) | 1.97 (1.30,2.41) | 1.59 (1.22,2.48) | 0.153 | 0.948 | 0.642 |
| *Inflammation* | | | | | | | | |
| NF-κB1 mRNA (AU) | 1.35 (1.08,1.62) | 1.41 (1.22,1.60) | 1.53 (1.27,1.97) | 1.33 (1.00,1.67) | 1.04 (0.80,1.30) | 0.417 | **0.040** | 0.087 |

| | | | | | | | | |
|---|---|---|---|---|---|---|---|---|
| TNF-α mRNA (AU) | 1.07 (0.73,1.16) | 1.07 (0.85, 1.31) | 1.31 (0.93, 1.94) | 0.80 (0.56,1.15) | 0.80 (0.46,1.38) | 0.870 | **0.026** | 0.301 |
| Catalase mRNA (AU) | 1.78 ± 0.53 | 1.72 ± 0.54 | 1.56 ±0.47 | 1.72 ± 0.57 | 1.70 ± 0.57 | 0.324 | 0.739 | 0.426 |

Normally distributed data reported as Mean ± Standard Deviation and skewed data reported as Median (Interquartile Range; IQR). Paired t-tests identified differences at baseline between depots (n=37). Repeated-measures ANOVA identified main effects of time (pre and post) and group (exercise and control), and interaction (group x time) effects reported for exercise (n=18 in abdominal and n=19 in gluteal depots) and control (n=14, both depots) groups. Difference between depots at baseline ^ <0.05. Glut4, Glucose transporter 4; IRS1, Insulin receptor substrate 1; PI3K, Phosphoinositide 3-Kinase; ATGL, Adipose triglyceride lipase; Lipoprotein lipase; DGAT1, Diacylglycerol O-acyltransferase; PPAR, Peroxisome proliferator-activated receptors.

**Supplementary Figure 1.**

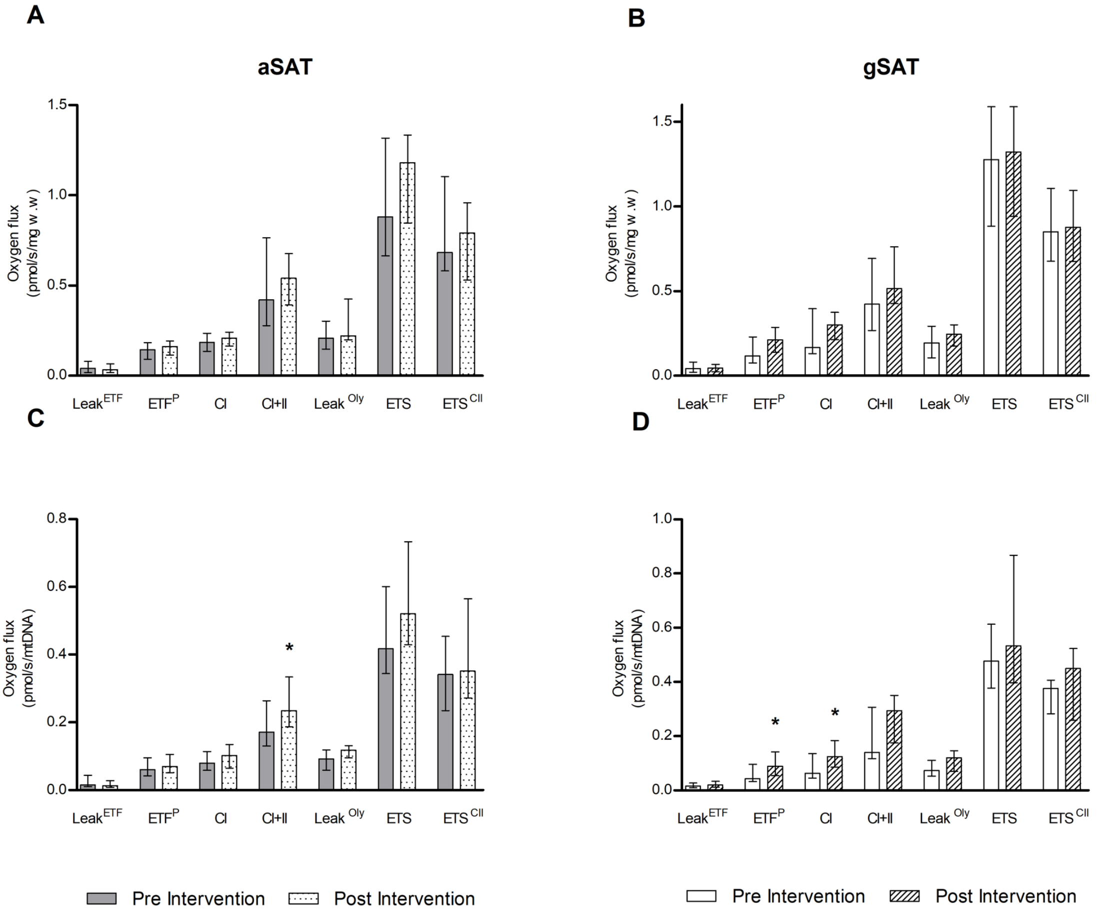


**Supplementary Figure 1:** Change in mitochondrial respiration in the control group over the 12-week intervention. A, C and E represent change in abdominal subcutaneous adipose tissue (aSAT). B, D and F represent change in gluteal SAT (gSAT). Mixed-model analyses identified main effects of time (pre and post) and group (exercise and control) and interaction (group x time) effects in exercise (n=19, both depots) and control (n=14 in abdominal and n=13 in gluteal depots) groups. All data reported as median (interquartile range). Significant difference between pre and post intervention in control group *$p<0.05$. Leak$^{ETF}$, leak respiration through electron-transferring flavoprotein; ETF$^{P}$, Lipid oxidative phosphorylation capacity; CI, Complex 1 linked respiration; CI+CII, Complex 1 and 2 linked respiration (oxidative phosphorylation capacity); Leak$^{Oly}$, Oligomycin (ATP synthase inhibitor) linked leak respiration; ETS, Electron transfer system capacity; ETS$^{II}$, Complex 2 linked electron transfer system capacity.

**Supplementary Figure 2.**

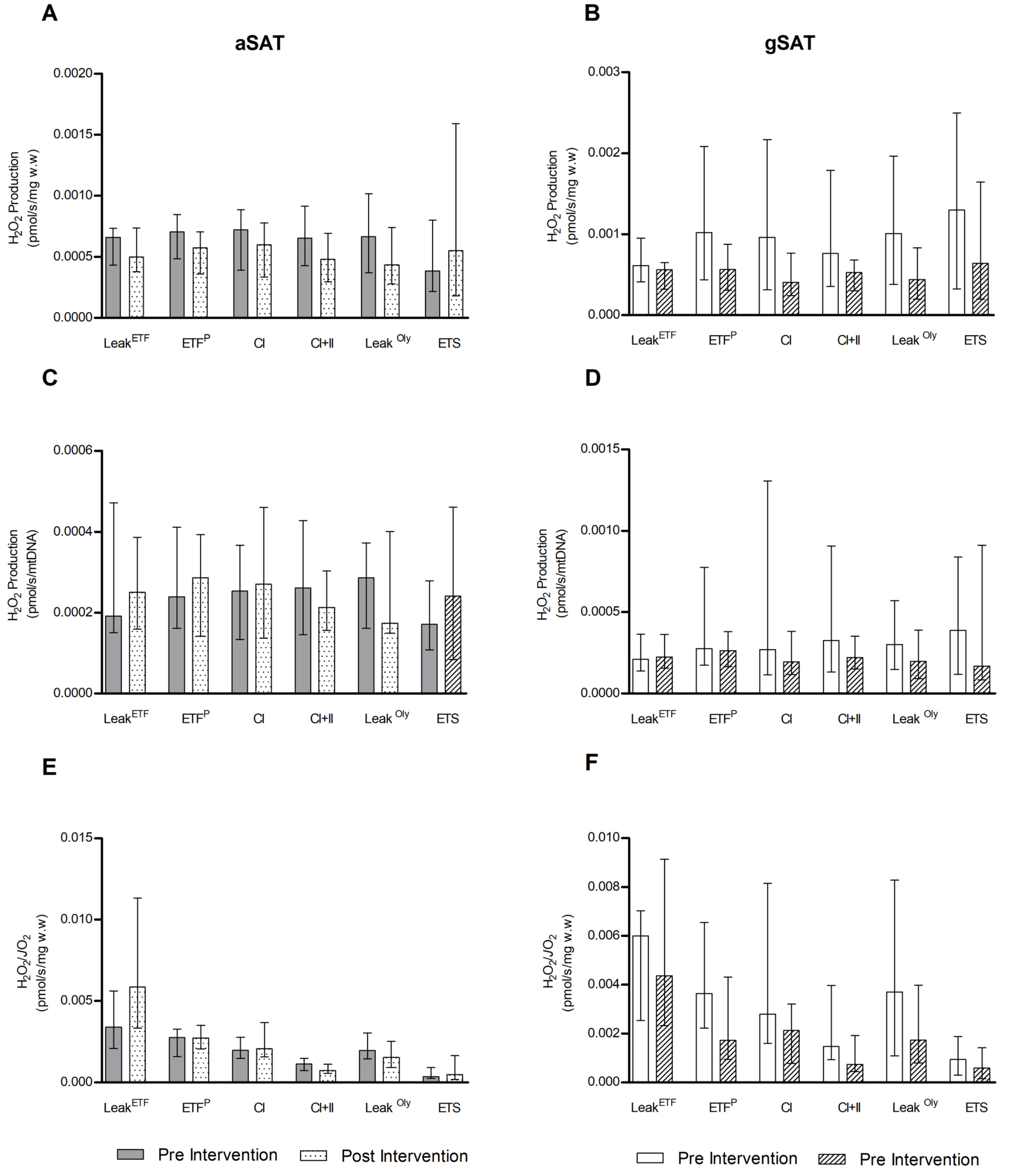


**Supplementary Figure 2:** Change in mitochondrial $H_2O_2$ production in the control group over the 12-week intervention. A, C and E represent change in abdominal subcutaneous adipose tissue (aSAT). B, D and F represent change in gluteal SAT (gSAT). Mixed-model analyses identified main effects of time (pre and post) and group (exercise and control) and interaction (group x time) effects in exercise (n=19, both depots) and control (n=14 in abdominal and n=13 in gluteal depots) groups. All data reported as median (interquartile range). No significant differences shown between over the intervention in the control group. $Leak^{ETF}$, leak respiration through electron-transferring flavoprotein; $ETF^{P}$, Lipid oxidative phosphorylation capacity; CI, Complex 1 linked respiration; CI+CII, Complex 1 and 2 linked respiration (oxidative phosphorylation capacity); $Leak^{Oly}$, Oligomycin (ATP synthase inhibitor) linked leak respiration; ETS, Electron transfer system capacity.
